# Crystal structure of *S*-hexyl (*E*)-3-(2-hy­droxy­benzyl­idene)di­thio­carbazate

**DOI:** 10.1107/S2056989016001857

**Published:** 2016-02-06

**Authors:** M. S. Begum, M. B. H. Howlader, M. C. Sheikh, R. Miyatake, E. Zangrando

**Affiliations:** a Department of Chemistry, Shahjalal University of Science and Technology, Sylhet 3114, Bangladesh; b Department of Chemistry, Rajshahi University, Rajshahi 6205, Bangladesh; c Department of Applied Chemistry, Faculty of Engineering, University of Toyama, 3190 Gofuku, Toyama 930-8555, Japan; d Center for Environmental Conservation and Research Safety, University of Toyama, 3190 Gofuku, Toyama 930-8555, Japan; e Department of Chemical and Pharmaceutical Sciences, via Giorgieri 1, 34127 Trieste, Italy

**Keywords:** crystal structure, di­thio­carbazate, S-containing Schiff base, tridentate ligand, hydrogen bonding

## Abstract

The title compound, C_14_H_20_N_2_OS_2_ [systematic name: *S*-hexyl (*E*)-2-(2-hy­droxy­benzyl­idene)hydrazine-1-carbodi­thio­ate], crystallizes with four independent mol­ecules (*A*–*D*) in the asymmetric unit. All four mol­ecules adopt an *E* conformation with respect to the C=N bond of the benzyl­idene moiety and have an intra­molecular O—H⋯N hydrogen bond generating an *S*(6) ring motif. In the crystal, the *A* and *D* mol­ecules are connected by a pair N—H⋯S hydrogen bonds, forming a dimer with an *R*_2_^2^(8) ring motif. In the case of mol­ecules *B* and *C*, they are linked to themselves by pairs of N—H⋯S hydrogen bonds, forming *B*–*B* and *C*–*C* inversion dimers with *R*_2_^2^(8) ring motifs.

## Abstract 2

The title compound crystallizes with four independent mol­ecules in the asymmetric unit, which have very comparable geometries. In the crystal, mol­ecules are connected in pairs through N—H⋯S hydrogen bonds, forming dimers.

## Chemical context   

Bidentate Schiff bases of S-methyl or S-benzyl di­thio­carbaza­tes and their metal complexes have received considerable attention for their possible bioactivities (Chan *et al.*, 2008 ▸; How *et al.*, 2008 ▸; Zangrando *et al.*, 2015 ▸; Ali *et al.*, 2002 ▸; Chew *et al.*, 2004 ▸; Crouse *et al.*, 2004 ▸). As part of our ongoing structural studies of S-containing Schiff bases, we report herein on the structure of a mol­ecule having a hexyl chain, similar to other ligands reported by our group (Begum, Zangrando *et al.*, 2015 ▸; Begum, Howlader, Miyatake *et al.*, 2015 ▸; Howlader *et al.*, 2015 ▸) but differing in their ability to act as tridentate ligands in metal coordination (Begum, Howlader, Sheikh *et al.*, 2015 ▸).
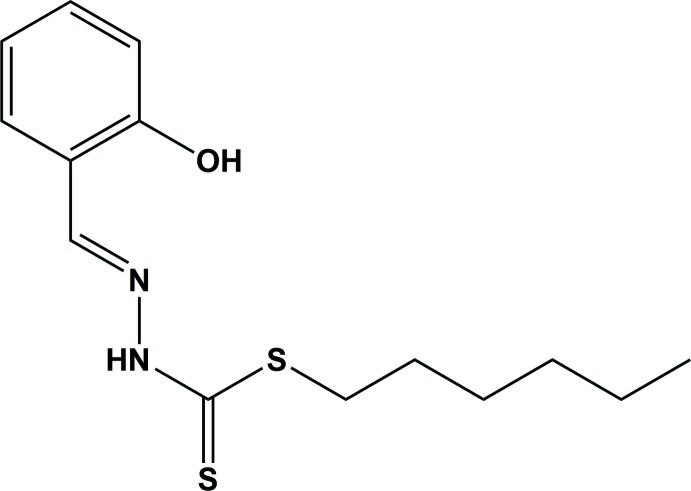


## Structural commentary   

**Figure 1:**
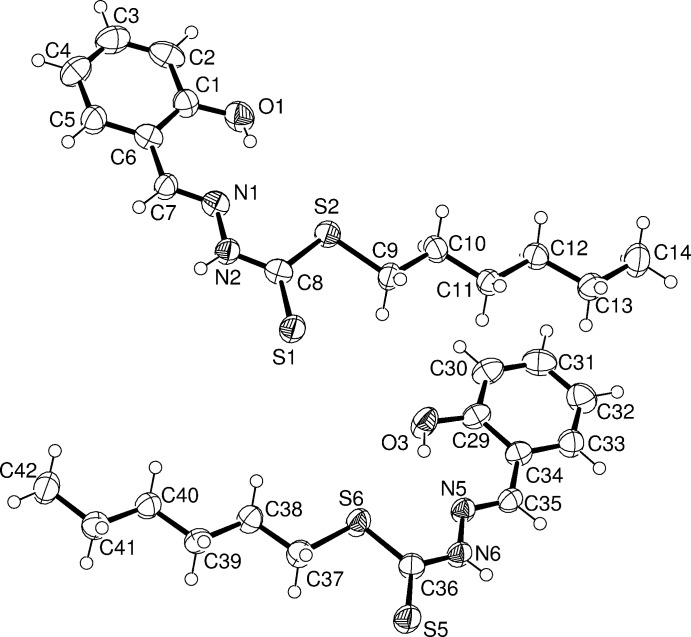
The mol­ecular structure of mol­ecules *A* and *C* of the title compound, showing the atom labelling. Displacement ellipsoids are drawn at the 50% probability level.

The four independent mol­ecules (*A-*-*D*) of the title compound are shown in Figs. 1 ▸ and 2 ▸. The Schiff base exists in its thione tautomeric form with the di­thio­carbazate fragment adopting an *E* conformation with respect to the C=N bond of the benzyl­idene moiety. The β-nitro­gen and the thio­keto sulfur are *trans* located with respect to bond C8—N2 bond in mol­ecule *A* (and similarly for mol­ecules *B*, *C* and *D*). All non-H atoms in the mol­ecules are almost co-planar indicating, except for the alkyl chain, electron delocalization within them. The maximum deviation from the mean plane is shown by the thio­ketone atoms S1, S3, S5 and S7 in the four independent mol­ecules (r.m.s deviations of 0.086, 0.118, 0.138 and 0.183 Å, respectively). The bond lengths and angles are comparable to those reported for *S*-hexyl (*E*)-3-(4-methyl­benzyl­idene)di­thio­carbazate (Howlader *et al.*, 2015 ▸) and *S*-hexyl (*E*)-3-(4-meth­oxy­benzyl­idene)di­thio­carbazate (Begum, Howlader, Miyatake *et al.*, 2015 ▸). The hexyl chain in all four mol­ecules has an extended *anti*-zigzag conformation. This compound in its deprotonated imino thiol­ate form has been reported to act as a tridentate ligand through N-, S- and O-donors to form a binuclear copper(II) complex (Begum, Howlader, Sheikh *et al.*, 2015 ▸).

## Supra­molecular features   

**Figure 3:**
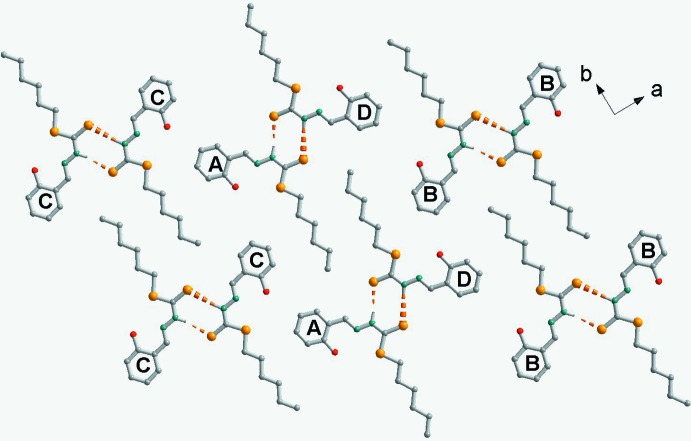
Crystal packing of the title compound, viewed along the *c* axis, showing pairs of mol­ecules connected by N—H.·S hydrogen bonds (dashed lines; see Table 1 ▸). H atoms not involved in hydrogen bonds have been omitted for clarity.

The crystal packing of the title compound (Fig. 3 ▸), indicates that the mol­ecules are connected by pairs of N—H⋯S hydrogen bonds (Table 1 ▸) to form *A*–*D* dimers, and *B*–*B* and *C*–*C* inversion dimers, all with 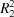
(8) ring motifs.

## Synthesis and crystallization   

To an ethano­lic solution of KOH (2.81 g, 0.05 mol), hydrazine hydrate (2.50 g, 0.05 mol, 99%) was added and the mixture stirred at 273 K. To this solution carbon di­sulfide (3.81 g, 0.05 mol) was added drop wise with constant stirring for 1 h. Then, 1-bromo­hexane (8.25 g, 0.05 mol) was added drop wise with vigorous stirring at 273 K for an additional hour. Finally, 2-hy­droxy­benzaldehyde (6.10 g, 0.05 mol) in ethanol was added and the mixture refluxed for 30 min. The mixture was filtered while hot and then the filtrate was cooled to 273 K giving a precipitate of the Schiff base product. It was recrystallized from ethanol at room temperature and dried in a vacuum desiccator over anhydrous CaCl_2_. Colourless crystals of the title compound were obtained by slow evaporation of a solution in methanol/aceto­nitrile (3:1) [m.p. 364 K].

## Refinement   

Crystal data, data collection and structure refinement details are summarized in Table 2 ▸. Hydrogen atoms were located geometrically and treated as riding atoms: C—H = 0.95–0.99 Å with *U*_iso_(H) = 1.2*U*_eq_(C). The hydrogen atoms of NH and OH groups were located in a difference Fourier map and refined with U_iso_(H) = 1.2U_eq_(N,O).

## Supplementary Material

Crystal structure: contains datablock(s) General, I. DOI: 10.1107/S2056989016001857/su5276sup1.cif

Structure factors: contains datablock(s) I. DOI: 10.1107/S2056989016001857/su5276Isup2.hkl

Supporting information file. DOI: 10.1107/S2056989016001857/su5276Isup3.cml

CCDC reference: 1451068

Additional supporting information:  crystallographic information; 3D view; checkCIF report

## Figures and Tables

**Figure 2:**
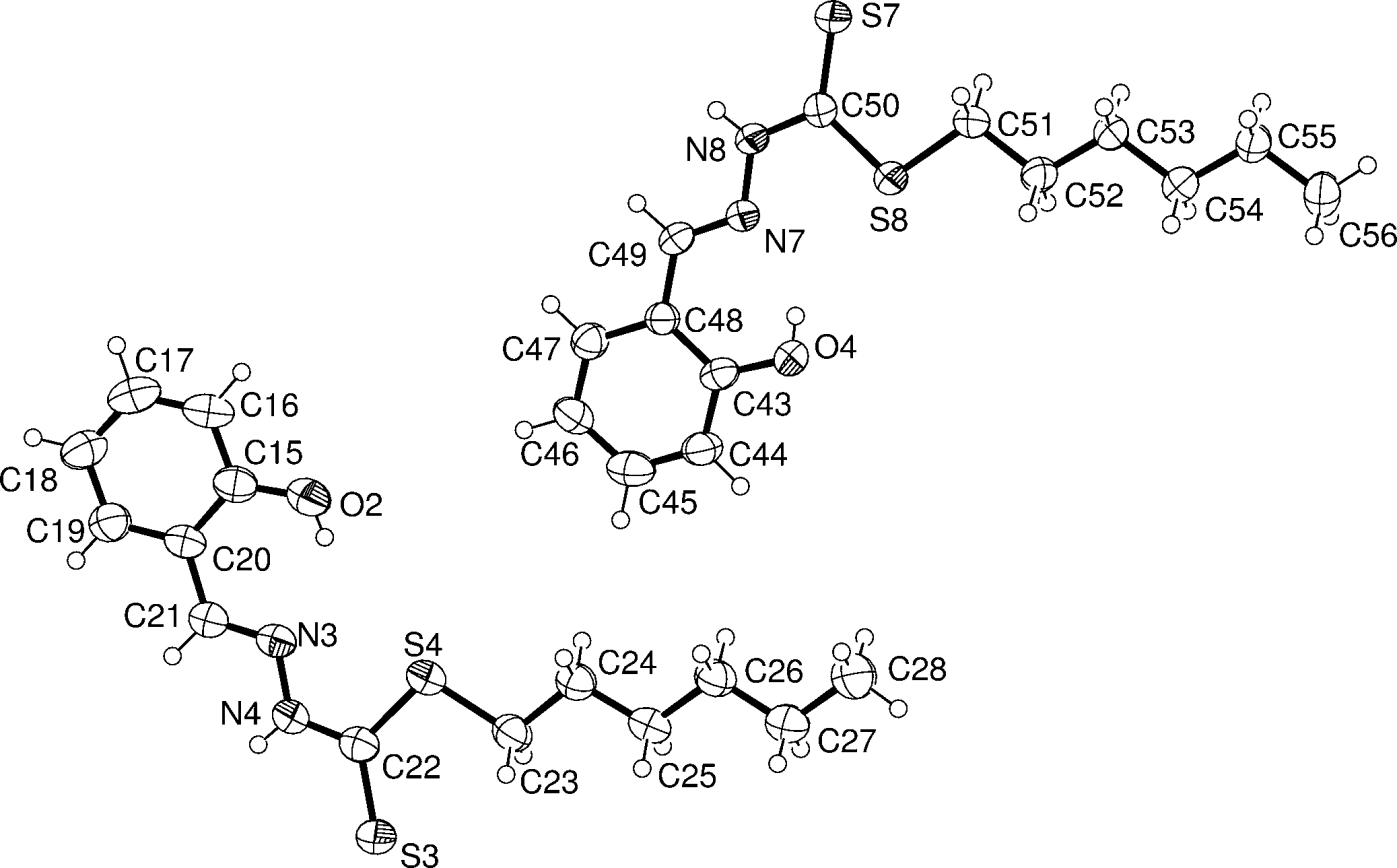
The mol­ecular structure of mol­ecules *B* and *D* of the title compound, showing the atom labelling. Displacement ellipsoids are drawn at the 50% probability level.

**Table 1:** Hydrogen-bond geometry (Å, °)

*D*—H⋯*A*	*D*—H	H⋯*A*	*D*⋯*A*	*D*—H⋯*A*
O1—H1*O*⋯N1	0.73 (3)	2.13 (3)	2.684 (3)	135 (3)
O2—H2*O*⋯N3	0.78 (3)	2.09 (3)	2.685 (3)	133 (3)
O3—H3*O*⋯N5	0.84 (3)	1.91 (3)	2.662 (3)	148 (3)
O4—H4*O*⋯N7	0.75 (3)	2.00 (3)	2.663 (3)	146 (3)
N2—H2*N*⋯S7^i^	0.86 (3)	2.58 (3)	3.430 (3)	169 (3)
N4—H4*N*⋯S3^ii^	0.81 (3)	2.64 (3)	3.439 (3)	171 (2)
N6—H6*N*⋯S5^iii^	0.84 (3)	2.59 (3)	3.398 (3)	162 (2)
N8—H8*N*⋯S1^iv^	0.83 (3)	2.61 (3)	3.403 (3)	160 (2)

Symmetry codes: (i) 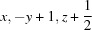; (ii) 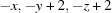; (iii) 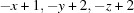; (iv) 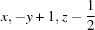.

**Table 2:** Experimental details

Crystal data	
Chemical formula	C_14_H_20_N_2_OS_2_
*M*_r_	296.44
Crystal system, space group	Monoclinic, *P*2/*c*
Temperature (K)	173
*a*, *b*, *c* (Å)	18.9744 (4), 16.0269 (3), 21.1146 (4)
β (°)	100.808 (1)
*V* (Å^3^)	6307.1 (2)
*Z*	16
Radiation type	Cu *K*α
μ (mm^−1^)	3.01
Crystal size (mm)	0.50 × 0.35 × 0.34

Data collection	
Diffractometer	Rigaku R-AXIS RAPID
No. of measured, independent and observed [*I* > 2σ(*I*)] reflections	10367, 10367, 6152
*R*_int_	0.072
(sin θ/λ)_max_ (Å^−1^)	0.581

Refinement	
*R*[*F* ^2^ > 2σ(*F* ^2^)], *wR*(*F* ^2^), *S*	0.065, 0.176, 0.93
No. of reflections	10367
No. of parameters	713
H-atom treatment	H-atom parameters constrained
Δρ_max_, Δρ_min_ (e Å^−3^)	1.02, −0.46

Computer programs: *RAPID-AUTO* (Rigaku, 2001 ▸), *SIR92* (Altomare *et al.*, 1994 ▸), *SHELXL2014* (Sheldrick, 2015 ▸) and *CrystalStructure* (Rigaku, 2010 ▸).

## supplementary crystallographic information

### Crystal data

**Table:**

C_14_H_20_N_2_OS_2_	*F*(000) = 2528
*M**_r_* = 296.44	*D*_x_ = 1.249 Mg m^−^^3^
Monoclinic, *P*2/*c*	Cu *K*α radiation, λ = 1.54187 Å
*a* = 18.9744 (4) Å	Cell parameters from 48491 reflections
*b* = 16.0269 (3) Å	θ = 3.5–68.2°
*c* = 21.1146 (4) Å	µ = 3.01 mm^−^^1^
β = 100.808 (1)°	*T* = 173 K
*V* = 6307.1 (2) Å^3^	Block, colourless
*Z* = 16	0.50 × 0.35 × 0.34 mm

### Data collection

**Table:**

Rigaku R-AXIS RAPID diffractometer	*R*_int_ = 0.072
Detector resolution: 10.000 pixels mm^-1^	θ_max_ = 63.7°, θ_min_ = 3.5°
ω scans	*h* = −22→22
10367 measured reflections	*k* = −19→19
10367 independent reflections	*l* = −25→25
6152 reflections with *I* > 2σ(*I*)	

### Refinement

**Table:**

Refinement on *F*^2^	Primary atom site location: structure-invariant direct methods
Least-squares matrix: full	Secondary atom site location: difference Fourier map
*R*[*F*^2^ > 2σ(*F*^2^)] = 0.065	Hydrogen site location: mixed
*wR*(*F*^2^) = 0.176	H-atom parameters constrained
*S* = 0.93	*w* = 1/[σ^2^(*F*_o_^2^) + (0.1083*P*)^2^] where *P* = (*F*_o_^2^ + 2*F*_c_^2^)/3
10367 reflections	(Δ/σ)_max_ = 0.001
713 parameters	Δρ_max_ = 1.01 e Å^−^^3^
0 restraints	Δρ_min_ = −0.46 e Å^−^^3^

### Special details

**Table:**

Geometry. All e.s.d.'s (except the e.s.d. in the dihedral angle between two l.s. planes) are estimated using the full covariance matrix. The cell e.s.d.'s are taken into account individually in the estimation of e.s.d.'s in distances, angles and torsion angles; correlations between e.s.d.'s in cell parameters are only used when they are defined by crystal symmetry. An approximate (isotropic) treatment of cell e.s.d.'s is used for estimating e.s.d.'s involving l.s. planes.

### Fractional atomic coordinates and isotropic or equivalent isotropic displacement parameters (Å^2^)

**Table:**

	*x*	*y*	*z*	*U*_iso_*/*U*_eq_	
S1	0.25358 (4)	0.59787 (5)	1.25919 (4)	0.0533 (2)	
S2	0.10299 (4)	0.66115 (5)	1.21116 (4)	0.0461 (2)	
O1	−0.06852 (11)	0.53508 (15)	1.15318 (11)	0.0557 (6)	
H1O	−0.0321 (17)	0.550 (2)	1.1640 (16)	0.067*	
N1	0.06892 (12)	0.49125 (15)	1.19459 (10)	0.0410 (6)	
N2	0.14075 (12)	0.50536 (15)	1.21817 (11)	0.0417 (6)	
H2N	0.1694 (15)	0.4634 (17)	1.2249 (13)	0.050*	
C1	−0.08129 (16)	0.4524 (2)	1.14951 (13)	0.0447 (7)	
C2	−0.15168 (17)	0.4254 (2)	1.13090 (14)	0.0570 (9)	
H2	−0.1893	0.4651	1.1212	0.068*	
C3	−0.16709 (17)	0.3420 (2)	1.12649 (15)	0.0617 (9)	
H3	−0.2155	0.3247	1.1136	0.074*	
C4	−0.11444 (17)	0.2829 (2)	1.14023 (14)	0.0584 (9)	
H4	−0.1261	0.2252	1.1369	0.070*	
C5	−0.04406 (16)	0.30837 (19)	1.15911 (13)	0.0508 (8)	
H5	−0.0073	0.2675	1.1688	0.061*	
C6	−0.02564 (15)	0.39267 (19)	1.16431 (12)	0.0418 (7)	
C7	0.04852 (14)	0.41577 (18)	1.18721 (12)	0.0409 (7)	
H7	0.0833	0.3728	1.1971	0.049*	
C8	0.16649 (15)	0.58236 (18)	1.22957 (13)	0.0410 (7)	
C9	0.15769 (15)	0.75494 (17)	1.22512 (14)	0.0445 (7)	
H9A	0.1819	0.7579	1.2709	0.053*	
H9B	0.1949	0.7542	1.1979	0.053*	
C10	0.10842 (14)	0.83027 (17)	1.20813 (13)	0.0434 (7)	
H10A	0.0840	0.8264	1.1625	0.052*	
H10B	0.0712	0.8300	1.2353	0.052*	
C11	0.15059 (15)	0.91139 (17)	1.21864 (13)	0.0420 (7)	
H11A	0.1756	0.9143	1.2642	0.050*	
H11B	0.1875	0.9115	1.1911	0.050*	
C12	0.10336 (14)	0.98812 (18)	1.20317 (13)	0.0437 (7)	
H12A	0.0643	0.9857	1.2283	0.052*	
H12B	0.0811	0.9871	1.1568	0.052*	
C13	0.14401 (15)	1.06987 (18)	1.21809 (14)	0.0474 (8)	
H13A	0.1664	1.0710	1.2644	0.057*	
H13B	0.1830	1.0726	1.1928	0.057*	
C14	0.09630 (16)	1.14567 (19)	1.20269 (16)	0.0633 (10)	
H14A	0.0737	1.1448	1.1570	0.095*	
H14B	0.1252	1.1964	1.2119	0.095*	
H14C	0.0591	1.1449	1.2292	0.095*	
S3	−0.00414 (4)	0.86442 (5)	0.98657 (4)	0.0615 (3)	
S4	0.14581 (4)	0.80245 (5)	1.04021 (4)	0.0527 (2)	
O2	0.31627 (12)	0.92929 (17)	1.09819 (11)	0.0628 (7)	
H2O	0.2772 (18)	0.913 (2)	1.0877 (17)	0.075*	
N3	0.17915 (13)	0.97231 (16)	1.05390 (10)	0.0453 (6)	
N4	0.10796 (14)	0.95825 (16)	1.02840 (12)	0.0483 (7)	
H4N	0.0804 (16)	0.997 (2)	1.0216 (14)	0.058*	
C15	0.32870 (17)	1.0119 (2)	1.10127 (13)	0.0511 (8)	
C16	0.39934 (17)	1.0388 (3)	1.12158 (15)	0.0620 (10)	
H16	0.4367	0.9990	1.1329	0.074*	
C17	0.41471 (19)	1.1219 (3)	1.12517 (15)	0.0678 (10)	
H17	0.4632	1.1390	1.1384	0.081*	
C18	0.36200 (18)	1.1823 (2)	1.11015 (15)	0.0640 (10)	
H18	0.3736	1.2400	1.1139	0.077*	
C19	0.29206 (18)	1.1563 (2)	1.08951 (14)	0.0581 (9)	
H19	0.2555	1.1970	1.0784	0.070*	
C20	0.27352 (16)	1.0720 (2)	1.08446 (13)	0.0461 (8)	
C21	0.19920 (16)	1.0485 (2)	1.06038 (13)	0.0467 (8)	
H21	0.1644	1.0912	1.0491	0.056*	
C22	0.08232 (16)	0.8808 (2)	1.01810 (14)	0.0484 (8)	
C23	0.09183 (16)	0.70822 (19)	1.02657 (14)	0.0519 (8)	
H23A	0.0551	0.7084	1.0543	0.062*	
H23B	0.0670	0.7055	0.9810	0.062*	
C24	0.14087 (15)	0.63370 (19)	1.04250 (14)	0.0509 (8)	
H24A	0.1650	0.6365	1.0883	0.061*	
H24B	0.1783	0.6350	1.0156	0.061*	
C25	0.09880 (15)	0.55256 (19)	1.03051 (14)	0.0516 (8)	
H25A	0.0633	0.5504	1.0594	0.062*	
H25B	0.0720	0.5522	0.9855	0.062*	
C26	0.14574 (16)	0.4750 (2)	1.04161 (15)	0.0540 (8)	
H26A	0.1696	0.4730	1.0875	0.065*	
H26B	0.1836	0.4791	1.0152	0.065*	
C27	0.10385 (17)	0.3945 (2)	1.02465 (16)	0.0613 (9)	
H27A	0.0652	0.3912	1.0502	0.074*	
H27B	0.0810	0.3960	0.9785	0.074*	
C28	0.15007 (18)	0.3170 (2)	1.03720 (17)	0.0776 (11)	
H28A	0.1879	0.3192	1.0114	0.116*	
H28B	0.1203	0.2674	1.0253	0.116*	
H28C	0.1719	0.3143	1.0830	0.116*	
S5	0.50812 (4)	0.86393 (5)	1.02000 (4)	0.0560 (3)	
S6	0.36038 (4)	0.79983 (5)	0.96181 (4)	0.0481 (2)	
O3	0.19255 (11)	0.91698 (14)	0.90094 (10)	0.0553 (6)	
H3O	0.2367 (16)	0.913 (2)	0.9149 (15)	0.066*	
N5	0.32576 (12)	0.96786 (14)	0.94728 (10)	0.0384 (6)	
N6	0.39644 (12)	0.95587 (15)	0.97387 (11)	0.0425 (6)	
H6N	0.4245 (15)	0.9962 (19)	0.9840 (13)	0.051*	
C29	0.17628 (15)	0.9992 (2)	0.89769 (12)	0.0433 (7)	
C30	0.10555 (16)	1.0218 (2)	0.87630 (14)	0.0541 (8)	
H30	0.0703	0.9797	0.8640	0.065*	
C31	0.08572 (17)	1.1037 (2)	0.87262 (14)	0.0573 (9)	
H31	0.0365	1.1176	0.8590	0.069*	
C32	0.13576 (17)	1.1674 (2)	0.88835 (14)	0.0565 (9)	
H32	0.1215	1.2243	0.8848	0.068*	
C33	0.20673 (16)	1.14573 (19)	0.90922 (13)	0.0476 (8)	
H33	0.2414	1.1886	0.9202	0.057*	
C34	0.22898 (15)	1.06259 (19)	0.91474 (12)	0.0399 (7)	
C35	0.30375 (15)	1.04327 (18)	0.94067 (12)	0.0397 (7)	
H35	0.3368	1.0875	0.9529	0.048*	
C36	0.42271 (15)	0.87873 (19)	0.98566 (13)	0.0428 (7)	
C37	0.41480 (15)	0.70616 (18)	0.97630 (14)	0.0460 (7)	
H37A	0.4535	0.7076	0.9508	0.055*	
H37B	0.4370	0.7020	1.0225	0.055*	
C38	0.36595 (15)	0.63222 (17)	0.95638 (13)	0.0446 (7)	
H38A	0.3448	0.6369	0.9100	0.053*	
H38B	0.3262	0.6332	0.9808	0.053*	
C39	0.40588 (14)	0.54952 (17)	0.96853 (13)	0.0433 (7)	
H39A	0.4275	0.5451	1.0149	0.052*	
H39B	0.4453	0.5483	0.9437	0.052*	
C40	0.35648 (15)	0.47508 (18)	0.94918 (14)	0.0456 (7)	
H40A	0.3358	0.4792	0.9026	0.055*	
H40B	0.3164	0.4775	0.9731	0.055*	
C41	0.39454 (16)	0.39093 (18)	0.96230 (15)	0.0525 (8)	
H41A	0.4325	0.3868	0.9361	0.063*	
H41B	0.4179	0.3881	1.0083	0.063*	
C42	0.34353 (17)	0.31711 (19)	0.94668 (16)	0.0638 (9)	
H42A	0.3058	0.3208	0.9725	0.096*	
H42B	0.3703	0.2650	0.9567	0.096*	
H42C	0.3218	0.3182	0.9008	0.096*	
S7	0.24114 (4)	0.67360 (5)	0.72575 (4)	0.0532 (2)	
S8	0.38731 (4)	0.73717 (5)	0.78991 (4)	0.0467 (2)	
O4	0.55528 (11)	0.61947 (14)	0.85012 (11)	0.0557 (6)	
H4O	0.5165 (16)	0.625 (2)	0.8343 (15)	0.067*	
N7	0.42251 (12)	0.56890 (14)	0.80139 (10)	0.0384 (6)	
N8	0.35164 (13)	0.58122 (15)	0.77368 (11)	0.0406 (6)	
H8N	0.3267 (15)	0.5403 (17)	0.7596 (13)	0.049*	
C43	0.57105 (15)	0.5377 (2)	0.85318 (12)	0.0423 (7)	
C44	0.64194 (16)	0.5149 (2)	0.87552 (14)	0.0518 (8)	
H44	0.6771	0.5569	0.8880	0.062*	
C45	0.66183 (17)	0.4330 (2)	0.87981 (15)	0.0579 (9)	
H45	0.7107	0.4190	0.8952	0.069*	
C46	0.61212 (17)	0.3694 (2)	0.86216 (14)	0.0534 (8)	
H46	0.6265	0.3125	0.8652	0.064*	
C47	0.54122 (16)	0.39123 (19)	0.84007 (13)	0.0465 (8)	
H47	0.5066	0.3485	0.8283	0.056*	
C48	0.51916 (15)	0.47465 (18)	0.83465 (12)	0.0384 (7)	
C49	0.44460 (14)	0.49335 (18)	0.80773 (12)	0.0385 (7)	
H49	0.4119	0.4490	0.7947	0.046*	
C50	0.32545 (15)	0.65816 (17)	0.76239 (13)	0.0406 (7)	
C51	0.33330 (15)	0.83076 (17)	0.77494 (14)	0.0448 (7)	
H51A	0.3105	0.8341	0.7288	0.054*	
H51B	0.2950	0.8301	0.8010	0.054*	
C52	0.38231 (15)	0.90513 (18)	0.79341 (13)	0.0445 (7)	
H52A	0.4035	0.9018	0.8399	0.053*	
H52B	0.4220	0.9030	0.7690	0.053*	
C53	0.34310 (14)	0.98712 (17)	0.77993 (13)	0.0424 (7)	
H53A	0.3051	0.9904	0.8061	0.051*	
H53B	0.3196	0.9889	0.7339	0.051*	
C54	0.39254 (15)	1.06271 (19)	0.79491 (14)	0.0503 (8)	
H54A	0.4157	1.0610	0.8410	0.060*	
H54B	0.4308	1.0590	0.7690	0.060*	
C55	0.35385 (16)	1.14549 (18)	0.78102 (16)	0.0558 (8)	
H55A	0.3290	1.1464	0.7354	0.067*	
H55B	0.3170	1.1504	0.8083	0.067*	
C56	0.40366 (17)	1.2195 (2)	0.79334 (17)	0.0731 (11)	
H56A	0.4289	1.2187	0.8383	0.110*	
H56B	0.3758	1.2711	0.7851	0.110*	
H56C	0.4386	1.2168	0.7646	0.110*	

### Atomic displacement parameters (Å^2^)

**Table:**

	*U*^11^	*U*^22^	*U*^33^	*U*^12^	*U*^13^	*U*^23^
S1	0.0338 (4)	0.0394 (5)	0.0820 (6)	0.0012 (3)	−0.0012 (4)	−0.0004 (4)
S2	0.0352 (4)	0.0406 (5)	0.0594 (5)	0.0030 (3)	0.0010 (4)	0.0001 (3)
O1	0.0457 (14)	0.0542 (16)	0.0627 (15)	0.0107 (12)	−0.0012 (12)	0.0022 (11)
N1	0.0363 (14)	0.0479 (16)	0.0359 (13)	0.0039 (12)	−0.0004 (10)	0.0023 (11)
N2	0.0328 (15)	0.0369 (16)	0.0521 (15)	0.0001 (11)	−0.0009 (12)	0.0001 (12)
C1	0.0446 (19)	0.0429 (19)	0.0439 (17)	0.0016 (15)	0.0013 (14)	−0.0036 (14)
C2	0.0351 (19)	0.085 (3)	0.0477 (19)	0.0163 (18)	−0.0011 (15)	0.0004 (17)
C3	0.043 (2)	0.079 (3)	0.060 (2)	−0.012 (2)	0.0005 (16)	−0.0065 (19)
C4	0.050 (2)	0.062 (2)	0.059 (2)	−0.0170 (18)	−0.0012 (16)	−0.0047 (17)
C5	0.0436 (19)	0.0424 (19)	0.063 (2)	−0.0008 (15)	0.0013 (16)	−0.0044 (15)
C6	0.0374 (17)	0.052 (2)	0.0343 (15)	0.0055 (15)	0.0029 (13)	0.0006 (13)
C7	0.0365 (17)	0.0345 (17)	0.0490 (17)	−0.0019 (14)	0.0010 (14)	−0.0019 (13)
C8	0.0379 (17)	0.048 (2)	0.0360 (15)	0.0048 (14)	0.0049 (13)	0.0039 (13)
C9	0.0395 (17)	0.0316 (16)	0.0600 (18)	−0.0020 (13)	0.0028 (14)	−0.0030 (14)
C10	0.0420 (18)	0.0375 (18)	0.0498 (17)	0.0052 (14)	0.0063 (14)	−0.0042 (13)
C11	0.0392 (17)	0.0379 (18)	0.0473 (17)	0.0023 (14)	0.0042 (14)	−0.0006 (13)
C12	0.0376 (17)	0.0402 (18)	0.0507 (18)	0.0040 (14)	0.0014 (14)	0.0000 (14)
C13	0.0400 (18)	0.0416 (19)	0.0580 (19)	−0.0014 (14)	0.0026 (15)	−0.0004 (14)
C14	0.060 (2)	0.040 (2)	0.084 (2)	0.0006 (17)	0.0009 (19)	−0.0033 (17)
S3	0.0391 (5)	0.0544 (6)	0.0868 (6)	0.0014 (4)	0.0009 (4)	0.0056 (4)
S4	0.0424 (5)	0.0528 (6)	0.0598 (5)	0.0060 (4)	0.0017 (4)	0.0058 (4)
O2	0.0508 (15)	0.0648 (18)	0.0687 (16)	0.0107 (14)	0.0011 (13)	0.0046 (12)
N3	0.0386 (15)	0.0561 (18)	0.0402 (14)	0.0042 (13)	0.0048 (11)	0.0042 (12)
N4	0.0410 (17)	0.0464 (18)	0.0548 (16)	0.0038 (13)	0.0021 (13)	0.0060 (13)
C15	0.048 (2)	0.065 (2)	0.0386 (17)	0.0035 (18)	0.0051 (15)	−0.0020 (16)
C16	0.040 (2)	0.088 (3)	0.054 (2)	0.0099 (19)	0.0001 (16)	−0.0031 (19)
C17	0.049 (2)	0.094 (3)	0.057 (2)	−0.015 (2)	0.0015 (17)	−0.013 (2)
C18	0.053 (2)	0.070 (3)	0.066 (2)	−0.010 (2)	0.0029 (18)	−0.0135 (18)
C19	0.053 (2)	0.059 (2)	0.060 (2)	−0.0014 (18)	0.0036 (17)	−0.0041 (17)
C20	0.0395 (18)	0.059 (2)	0.0387 (16)	0.0057 (16)	0.0040 (14)	0.0013 (14)
C21	0.0424 (19)	0.053 (2)	0.0440 (17)	0.0034 (16)	0.0072 (14)	0.0014 (15)
C22	0.0444 (19)	0.056 (2)	0.0442 (17)	0.0032 (16)	0.0061 (14)	0.0035 (15)
C23	0.0451 (19)	0.054 (2)	0.0563 (19)	0.0072 (16)	0.0082 (15)	0.0068 (15)
C24	0.045 (2)	0.055 (2)	0.0524 (19)	0.0034 (16)	0.0092 (15)	0.0016 (15)
C25	0.0440 (19)	0.060 (2)	0.0486 (18)	0.0092 (17)	0.0041 (15)	0.0039 (15)
C26	0.045 (2)	0.057 (2)	0.059 (2)	0.0062 (17)	0.0073 (16)	0.0005 (16)
C27	0.048 (2)	0.065 (2)	0.070 (2)	0.0042 (18)	0.0062 (17)	0.0011 (18)
C28	0.073 (3)	0.062 (3)	0.093 (3)	0.005 (2)	0.004 (2)	−0.007 (2)
S5	0.0355 (5)	0.0451 (5)	0.0812 (6)	−0.0009 (4)	−0.0053 (4)	−0.0014 (4)
S6	0.0391 (5)	0.0375 (5)	0.0627 (5)	−0.0030 (3)	−0.0036 (4)	−0.0002 (3)
O3	0.0466 (13)	0.0426 (13)	0.0691 (15)	−0.0093 (11)	−0.0084 (12)	0.0045 (10)
N5	0.0343 (14)	0.0380 (15)	0.0404 (13)	−0.0031 (11)	0.0002 (11)	−0.0025 (10)
N6	0.0307 (15)	0.0404 (16)	0.0518 (15)	−0.0038 (11)	−0.0043 (12)	−0.0035 (12)
C29	0.0376 (18)	0.052 (2)	0.0373 (16)	−0.0075 (15)	−0.0005 (13)	0.0028 (14)
C30	0.0400 (19)	0.063 (2)	0.0559 (19)	−0.0093 (17)	−0.0001 (15)	0.0054 (16)
C31	0.0375 (19)	0.076 (3)	0.056 (2)	0.0070 (18)	0.0017 (15)	0.0084 (18)
C32	0.050 (2)	0.058 (2)	0.058 (2)	0.0107 (18)	0.0020 (16)	0.0035 (16)
C33	0.0447 (19)	0.043 (2)	0.0521 (18)	0.0003 (15)	0.0022 (15)	−0.0013 (14)
C34	0.0355 (17)	0.0462 (19)	0.0357 (15)	−0.0020 (14)	0.0010 (13)	0.0014 (13)
C35	0.0367 (17)	0.0412 (19)	0.0401 (16)	−0.0071 (14)	0.0045 (13)	−0.0032 (13)
C36	0.0389 (18)	0.0433 (19)	0.0444 (16)	0.0014 (14)	0.0030 (13)	−0.0003 (14)
C37	0.0410 (18)	0.0420 (18)	0.0518 (18)	0.0007 (14)	0.0008 (14)	0.0037 (14)
C38	0.0439 (18)	0.0402 (18)	0.0466 (17)	0.0000 (14)	0.0007 (14)	0.0054 (13)
C39	0.0371 (17)	0.047 (2)	0.0438 (17)	−0.0035 (14)	0.0030 (14)	0.0008 (14)
C40	0.0393 (18)	0.0438 (19)	0.0519 (18)	−0.0031 (14)	0.0041 (14)	−0.0016 (14)
C41	0.0446 (19)	0.048 (2)	0.064 (2)	0.0002 (16)	0.0060 (16)	−0.0003 (16)
C42	0.058 (2)	0.043 (2)	0.088 (2)	−0.0056 (17)	0.0053 (19)	−0.0039 (17)
S7	0.0354 (5)	0.0434 (5)	0.0758 (6)	0.0005 (3)	−0.0025 (4)	0.0030 (4)
S8	0.0410 (5)	0.0362 (5)	0.0580 (5)	−0.0009 (3)	−0.0035 (4)	−0.0021 (3)
O4	0.0502 (14)	0.0400 (13)	0.0693 (15)	−0.0074 (12)	−0.0082 (12)	0.0024 (11)
N7	0.0349 (14)	0.0360 (14)	0.0420 (13)	−0.0009 (11)	0.0015 (11)	−0.0006 (10)
N8	0.0346 (15)	0.0361 (16)	0.0475 (14)	−0.0028 (11)	−0.0016 (12)	−0.0048 (11)
C43	0.0374 (18)	0.054 (2)	0.0348 (15)	−0.0084 (15)	0.0040 (13)	0.0003 (13)
C44	0.0380 (18)	0.059 (2)	0.0554 (19)	−0.0058 (16)	0.0021 (15)	0.0042 (16)
C45	0.0395 (19)	0.083 (3)	0.0508 (19)	0.0036 (19)	0.0078 (15)	0.0061 (18)
C46	0.048 (2)	0.053 (2)	0.057 (2)	0.0119 (17)	0.0044 (16)	0.0045 (16)
C47	0.0459 (19)	0.046 (2)	0.0471 (17)	−0.0016 (15)	0.0068 (15)	−0.0045 (14)
C48	0.0350 (16)	0.0413 (17)	0.0385 (16)	−0.0005 (14)	0.0056 (13)	0.0019 (13)
C49	0.0366 (17)	0.0423 (18)	0.0357 (15)	−0.0079 (14)	0.0048 (13)	−0.0045 (13)
C50	0.0388 (17)	0.0366 (17)	0.0461 (16)	0.0003 (14)	0.0068 (13)	0.0013 (13)
C51	0.0382 (17)	0.0439 (19)	0.0482 (17)	0.0006 (14)	−0.0023 (14)	0.0003 (14)
C52	0.0456 (18)	0.0446 (19)	0.0408 (16)	−0.0044 (15)	0.0014 (14)	−0.0005 (13)
C53	0.0424 (18)	0.0390 (18)	0.0439 (16)	−0.0013 (14)	0.0031 (14)	−0.0011 (13)
C54	0.0434 (19)	0.044 (2)	0.061 (2)	−0.0043 (15)	0.0049 (16)	−0.0016 (15)
C55	0.046 (2)	0.0397 (19)	0.081 (2)	−0.0017 (15)	0.0100 (17)	−0.0015 (16)
C56	0.063 (2)	0.044 (2)	0.111 (3)	−0.0017 (18)	0.012 (2)	0.006 (2)

### Geometric parameters (Å, º)

**Table:**

S1—C8	1.672 (3)	S5—C36	1.664 (3)
S2—C8	1.738 (3)	S6—C36	1.740 (3)
S2—C9	1.819 (3)	S6—C37	1.815 (3)
O1—C1	1.347 (4)	O3—C29	1.353 (4)
O1—H1O	0.73 (3)	O3—H3O	0.84 (3)
N1—C7	1.271 (3)	N5—C35	1.278 (3)
N1—N2	1.379 (3)	N5—N6	1.367 (3)
N2—C8	1.332 (3)	N6—C36	1.339 (4)
N2—H2N	0.86 (3)	N6—H6N	0.84 (3)
C1—C2	1.389 (4)	C29—C30	1.381 (4)
C1—C6	1.416 (4)	C29—C34	1.423 (4)
C2—C3	1.368 (5)	C30—C31	1.364 (4)
C2—H2	0.9500	C30—H30	0.9500
C3—C4	1.368 (4)	C31—C32	1.392 (4)
C3—H3	0.9500	C31—H31	0.9500
C4—C5	1.382 (4)	C32—C33	1.380 (4)
C4—H4	0.9500	C32—H32	0.9500
C5—C6	1.395 (4)	C33—C34	1.396 (4)
C5—H5	0.9500	C33—H33	0.9500
C6—C7	1.448 (4)	C34—C35	1.455 (4)
C7—H7	0.9500	C35—H35	0.9500
C9—C10	1.528 (4)	C37—C38	1.515 (4)
C9—H9A	0.9900	C37—H37A	0.9900
C9—H9B	0.9900	C37—H37B	0.9900
C10—C11	1.521 (4)	C38—C39	1.524 (4)
C10—H10A	0.9900	C38—H38A	0.9900
C10—H10B	0.9900	C38—H38B	0.9900
C11—C12	1.521 (4)	C39—C40	1.525 (4)
C11—H11A	0.9900	C39—H39A	0.9900
C11—H11B	0.9900	C39—H39B	0.9900
C12—C13	1.523 (4)	C40—C41	1.530 (4)
C12—H12A	0.9900	C40—H40A	0.9900
C12—H12B	0.9900	C40—H40B	0.9900
C13—C14	1.513 (4)	C41—C42	1.525 (4)
C13—H13A	0.9900	C41—H41A	0.9900
C13—H13B	0.9900	C41—H41B	0.9900
C14—H14A	0.9800	C42—H42A	0.9800
C14—H14B	0.9800	C42—H42B	0.9800
C14—H14C	0.9800	C42—H42C	0.9800
S3—C22	1.672 (3)	S7—C50	1.660 (3)
S4—C22	1.742 (3)	S8—C50	1.750 (3)
S4—C23	1.817 (3)	S8—C51	1.811 (3)
O2—C15	1.344 (4)	O4—C43	1.343 (4)
O2—H2O	0.78 (3)	O4—H4O	0.76 (3)
N3—C21	1.278 (3)	N7—C49	1.280 (3)
N3—N4	1.376 (3)	N7—N8	1.376 (3)
N4—C22	1.336 (4)	N8—C50	1.334 (3)
N4—H4N	0.81 (3)	N8—H8N	0.83 (3)
C15—C16	1.397 (4)	C43—C44	1.388 (4)
C15—C20	1.418 (4)	C43—C48	1.414 (4)
C16—C17	1.363 (5)	C44—C45	1.364 (4)
C16—H16	0.9500	C44—H44	0.9500
C17—C18	1.385 (5)	C45—C46	1.391 (4)
C17—H17	0.9500	C45—H45	0.9500
C18—C19	1.381 (4)	C46—C47	1.384 (4)
C18—H18	0.9500	C46—H46	0.9500
C19—C20	1.395 (4)	C47—C48	1.399 (4)
C19—H19	0.9500	C47—H47	0.9500
C20—C21	1.456 (4)	C48—C49	1.453 (4)
C21—H21	0.9500	C49—H49	0.9500
C23—C24	1.513 (4)	C51—C52	1.517 (4)
C23—H23A	0.9900	C51—H51A	0.9900
C23—H23B	0.9900	C51—H51B	0.9900
C24—C25	1.522 (4)	C52—C53	1.511 (4)
C24—H24A	0.9900	C52—H52A	0.9900
C24—H24B	0.9900	C52—H52B	0.9900
C25—C26	1.521 (4)	C53—C54	1.529 (4)
C25—H25A	0.9900	C53—H53A	0.9900
C25—H25B	0.9900	C53—H53B	0.9900
C26—C27	1.522 (4)	C54—C55	1.518 (4)
C26—H26A	0.9900	C54—H54A	0.9900
C26—H26B	0.9900	C54—H54B	0.9900
C27—C28	1.515 (4)	C55—C56	1.509 (4)
C27—H27A	0.9900	C55—H55A	0.9900
C27—H27B	0.9900	C55—H55B	0.9900
C28—H28A	0.9800	C56—H56A	0.9800
C28—H28B	0.9800	C56—H56B	0.9800
C28—H28C	0.9800	C56—H56C	0.9800
			
C8—S2—C9	102.33 (13)	C36—S6—C37	102.60 (14)
C1—O1—H1O	119 (3)	C29—O3—H3O	107 (2)
C7—N1—N2	117.3 (2)	C35—N5—N6	116.9 (2)
C8—N2—N1	121.4 (2)	C36—N6—N5	120.6 (2)
C8—N2—H2N	119.8 (19)	C36—N6—H6N	118 (2)
N1—N2—H2N	118.8 (19)	N5—N6—H6N	122 (2)
O1—C1—C2	118.4 (3)	O3—C29—C30	118.0 (3)
O1—C1—C6	122.3 (3)	O3—C29—C34	122.7 (3)
C2—C1—C6	119.3 (3)	C30—C29—C34	119.3 (3)
C3—C2—C1	120.4 (3)	C31—C30—C29	120.7 (3)
C3—C2—H2	119.8	C31—C30—H30	119.6
C1—C2—H2	119.8	C29—C30—H30	119.6
C2—C3—C4	121.6 (3)	C30—C31—C32	121.6 (3)
C2—C3—H3	119.2	C30—C31—H31	119.2
C4—C3—H3	119.2	C32—C31—H31	119.2
C3—C4—C5	118.9 (3)	C33—C32—C31	118.2 (3)
C3—C4—H4	120.5	C33—C32—H32	120.9
C5—C4—H4	120.5	C31—C32—H32	120.9
C4—C5—C6	121.6 (3)	C32—C33—C34	121.9 (3)
C4—C5—H5	119.2	C32—C33—H33	119.0
C6—C5—H5	119.2	C34—C33—H33	119.0
C5—C6—C1	118.2 (3)	C33—C34—C29	118.2 (3)
C5—C6—C7	119.2 (3)	C33—C34—C35	119.5 (3)
C1—C6—C7	122.6 (3)	C29—C34—C35	122.2 (3)
N1—C7—C6	122.6 (3)	N5—C35—C34	121.2 (3)
N1—C7—H7	118.7	N5—C35—H35	119.4
C6—C7—H7	118.7	C34—C35—H35	119.4
N2—C8—S1	120.6 (2)	N6—C36—S5	120.7 (2)
N2—C8—S2	114.7 (2)	N6—C36—S6	114.1 (2)
S1—C8—S2	124.79 (18)	S5—C36—S6	125.18 (18)
C10—C9—S2	108.02 (19)	C38—C37—S6	107.53 (19)
C10—C9—H9A	110.1	C38—C37—H37A	110.2
S2—C9—H9A	110.1	S6—C37—H37A	110.2
C10—C9—H9B	110.1	C38—C37—H37B	110.2
S2—C9—H9B	110.1	S6—C37—H37B	110.2
H9A—C9—H9B	108.4	H37A—C37—H37B	108.5
C11—C10—C9	111.0 (2)	C37—C38—C39	112.0 (2)
C11—C10—H10A	109.4	C37—C38—H38A	109.2
C9—C10—H10A	109.4	C39—C38—H38A	109.2
C11—C10—H10B	109.4	C37—C38—H38B	109.2
C9—C10—H10B	109.4	C39—C38—H38B	109.2
H10A—C10—H10B	108.0	H38A—C38—H38B	107.9
C12—C11—C10	112.8 (2)	C38—C39—C40	112.0 (2)
C12—C11—H11A	109.0	C38—C39—H39A	109.2
C10—C11—H11A	109.0	C40—C39—H39A	109.2
C12—C11—H11B	109.0	C38—C39—H39B	109.2
C10—C11—H11B	109.0	C40—C39—H39B	109.2
H11A—C11—H11B	107.8	H39A—C39—H39B	107.9
C11—C12—C13	113.3 (2)	C39—C40—C41	113.3 (2)
C11—C12—H12A	108.9	C39—C40—H40A	108.9
C13—C12—H12A	108.9	C41—C40—H40A	108.9
C11—C12—H12B	108.9	C39—C40—H40B	108.9
C13—C12—H12B	108.9	C41—C40—H40B	108.9
H12A—C12—H12B	107.7	H40A—C40—H40B	107.7
C14—C13—C12	112.7 (2)	C42—C41—C40	112.7 (2)
C14—C13—H13A	109.0	C42—C41—H41A	109.1
C12—C13—H13A	109.0	C40—C41—H41A	109.1
C14—C13—H13B	109.0	C42—C41—H41B	109.1
C12—C13—H13B	109.0	C40—C41—H41B	109.1
H13A—C13—H13B	107.8	H41A—C41—H41B	107.8
C13—C14—H14A	109.5	C41—C42—H42A	109.5
C13—C14—H14B	109.5	C41—C42—H42B	109.5
H14A—C14—H14B	109.5	H42A—C42—H42B	109.5
C13—C14—H14C	109.5	C41—C42—H42C	109.5
H14A—C14—H14C	109.5	H42A—C42—H42C	109.5
H14B—C14—H14C	109.5	H42B—C42—H42C	109.5
C22—S4—C23	102.46 (15)	C50—S8—C51	102.66 (13)
C15—O2—H2O	119 (3)	C43—O4—H4O	109 (3)
C21—N3—N4	116.7 (3)	C49—N7—N8	117.1 (2)
C22—N4—N3	121.1 (3)	C50—N8—N7	120.7 (2)
C22—N4—H4N	119 (2)	C50—N8—H8N	120 (2)
N3—N4—H4N	120 (2)	N7—N8—H8N	119 (2)
O2—C15—C16	117.9 (3)	O4—C43—C44	117.8 (3)
O2—C15—C20	122.8 (3)	O4—C43—C48	123.1 (3)
C16—C15—C20	119.2 (3)	C44—C43—C48	119.1 (3)
C17—C16—C15	120.1 (3)	C45—C44—C43	121.0 (3)
C17—C16—H16	119.9	C45—C44—H44	119.5
C15—C16—H16	119.9	C43—C44—H44	119.5
C16—C17—C18	122.2 (3)	C44—C45—C46	121.5 (3)
C16—C17—H17	118.9	C44—C45—H45	119.3
C18—C17—H17	118.9	C46—C45—H45	119.3
C19—C18—C17	118.1 (3)	C47—C46—C45	118.2 (3)
C19—C18—H18	120.9	C47—C46—H46	120.9
C17—C18—H18	120.9	C45—C46—H46	120.9
C18—C19—C20	122.0 (3)	C46—C47—C48	121.8 (3)
C18—C19—H19	119.0	C46—C47—H47	119.1
C20—C19—H19	119.0	C48—C47—H47	119.1
C19—C20—C15	118.4 (3)	C47—C48—C43	118.6 (3)
C19—C20—C21	119.4 (3)	C47—C48—C49	118.9 (3)
C15—C20—C21	122.2 (3)	C43—C48—C49	122.5 (3)
N3—C21—C20	122.3 (3)	N7—C49—C48	120.8 (3)
N3—C21—H21	118.9	N7—C49—H49	119.6
C20—C21—H21	118.9	C48—C49—H49	119.6
N4—C22—S3	120.7 (2)	N8—C50—S7	121.0 (2)
N4—C22—S4	114.4 (2)	N8—C50—S8	113.9 (2)
S3—C22—S4	124.8 (2)	S7—C50—S8	125.07 (17)
C24—C23—S4	108.4 (2)	C52—C51—S8	107.84 (19)
C24—C23—H23A	110.0	C52—C51—H51A	110.1
S4—C23—H23A	110.0	S8—C51—H51A	110.1
C24—C23—H23B	110.0	C52—C51—H51B	110.1
S4—C23—H23B	110.0	S8—C51—H51B	110.1
H23A—C23—H23B	108.4	H51A—C51—H51B	108.5
C23—C24—C25	110.9 (2)	C53—C52—C51	112.2 (2)
C23—C24—H24A	109.5	C53—C52—H52A	109.2
C25—C24—H24A	109.5	C51—C52—H52A	109.2
C23—C24—H24B	109.5	C53—C52—H52B	109.2
C25—C24—H24B	109.5	C51—C52—H52B	109.2
H24A—C24—H24B	108.1	H52A—C52—H52B	107.9
C26—C25—C24	113.5 (2)	C52—C53—C54	112.9 (2)
C26—C25—H25A	108.9	C52—C53—H53A	109.0
C24—C25—H25A	108.9	C54—C53—H53A	109.0
C26—C25—H25B	108.9	C52—C53—H53B	109.0
C24—C25—H25B	108.9	C54—C53—H53B	109.0
H25A—C25—H25B	107.7	H53A—C53—H53B	107.8
C25—C26—C27	113.0 (3)	C55—C54—C53	113.4 (2)
C25—C26—H26A	109.0	C55—C54—H54A	108.9
C27—C26—H26A	109.0	C53—C54—H54A	108.9
C25—C26—H26B	109.0	C55—C54—H54B	108.9
C27—C26—H26B	109.0	C53—C54—H54B	108.9
H26A—C26—H26B	107.8	H54A—C54—H54B	107.7
C28—C27—C26	113.2 (3)	C56—C55—C54	112.9 (2)
C28—C27—H27A	108.9	C56—C55—H55A	109.0
C26—C27—H27A	108.9	C54—C55—H55A	109.0
C28—C27—H27B	108.9	C56—C55—H55B	109.0
C26—C27—H27B	108.9	C54—C55—H55B	109.0
H27A—C27—H27B	107.8	H55A—C55—H55B	107.8
C27—C28—H28A	109.5	C55—C56—H56A	109.5
C27—C28—H28B	109.5	C55—C56—H56B	109.5
H28A—C28—H28B	109.5	H56A—C56—H56B	109.5
C27—C28—H28C	109.5	C55—C56—H56C	109.5
H28A—C28—H28C	109.5	H56A—C56—H56C	109.5
H28B—C28—H28C	109.5	H56B—C56—H56C	109.5
			
C7—N1—N2—C8	−177.6 (2)	C35—N5—N6—C36	176.3 (2)
O1—C1—C2—C3	179.9 (3)	O3—C29—C30—C31	179.3 (3)
C6—C1—C2—C3	−0.4 (4)	C34—C29—C30—C31	−1.3 (4)
C1—C2—C3—C4	0.2 (5)	C29—C30—C31—C32	1.8 (5)
C2—C3—C4—C5	0.1 (5)	C30—C31—C32—C33	−1.2 (5)
C3—C4—C5—C6	−0.1 (5)	C31—C32—C33—C34	0.1 (4)
C4—C5—C6—C1	−0.1 (4)	C32—C33—C34—C29	0.5 (4)
C4—C5—C6—C7	177.1 (3)	C32—C33—C34—C35	−176.3 (3)
O1—C1—C6—C5	−180.0 (3)	O3—C29—C34—C33	179.5 (2)
C2—C1—C6—C5	0.4 (4)	C30—C29—C34—C33	0.1 (4)
O1—C1—C6—C7	2.9 (4)	O3—C29—C34—C35	−3.8 (4)
C2—C1—C6—C7	−176.8 (3)	C30—C29—C34—C35	176.8 (2)
N2—N1—C7—C6	178.5 (2)	N6—N5—C35—C34	−178.1 (2)
C5—C6—C7—N1	−178.3 (3)	C33—C34—C35—N5	179.0 (2)
C1—C6—C7—N1	−1.2 (4)	C29—C34—C35—N5	2.3 (4)
N1—N2—C8—S1	179.09 (18)	N5—N6—C36—S5	−178.17 (19)
N1—N2—C8—S2	−1.0 (3)	N5—N6—C36—S6	2.1 (3)
C9—S2—C8—N2	−174.8 (2)	C37—S6—C36—N6	174.1 (2)
C9—S2—C8—S1	5.1 (2)	C37—S6—C36—S5	−5.6 (2)
C8—S2—C9—C10	177.90 (19)	C36—S6—C37—C38	−179.37 (19)
S2—C9—C10—C11	−179.99 (19)	S6—C37—C38—C39	−178.21 (19)
C9—C10—C11—C12	−179.2 (2)	C37—C38—C39—C40	179.3 (2)
C10—C11—C12—C13	175.8 (2)	C38—C39—C40—C41	−178.6 (2)
C11—C12—C13—C14	−179.8 (2)	C39—C40—C41—C42	176.4 (2)
C21—N3—N4—C22	178.1 (3)	C49—N7—N8—C50	−176.6 (2)
O2—C15—C16—C17	179.7 (3)	O4—C43—C44—C45	−179.9 (3)
C20—C15—C16—C17	0.1 (4)	C48—C43—C44—C45	−0.1 (4)
C15—C16—C17—C18	1.0 (5)	C43—C44—C45—C46	−0.1 (5)
C16—C17—C18—C19	−1.5 (5)	C44—C45—C46—C47	−0.2 (4)
C17—C18—C19—C20	1.0 (5)	C45—C46—C47—C48	0.7 (4)
C18—C19—C20—C15	0.1 (4)	C46—C47—C48—C43	−0.8 (4)
C18—C19—C20—C21	−178.0 (3)	C46—C47—C48—C49	176.6 (2)
O2—C15—C20—C19	179.8 (3)	O4—C43—C48—C47	−179.7 (2)
C16—C15—C20—C19	−0.6 (4)	C44—C43—C48—C47	0.5 (4)
O2—C15—C20—C21	−2.2 (4)	O4—C43—C48—C49	3.0 (4)
C16—C15—C20—C21	177.4 (3)	C44—C43—C48—C49	−176.8 (2)
N4—N3—C21—C20	−177.6 (2)	N8—N7—C49—C48	178.1 (2)
C19—C20—C21—N3	179.1 (3)	C47—C48—C49—N7	−179.3 (2)
C15—C20—C21—N3	1.1 (4)	C43—C48—C49—N7	−1.9 (4)
N3—N4—C22—S3	−179.53 (19)	N7—N8—C50—S7	176.98 (18)
N3—N4—C22—S4	0.6 (4)	N7—N8—C50—S8	−3.4 (3)
C23—S4—C22—N4	175.2 (2)	C51—S8—C50—N8	−174.4 (2)
C23—S4—C22—S3	−4.7 (2)	C51—S8—C50—S7	5.2 (2)
C22—S4—C23—C24	178.7 (2)	C50—S8—C51—C52	−176.75 (19)
S4—C23—C24—C25	−178.71 (19)	S8—C51—C52—C53	177.41 (19)
C23—C24—C25—C26	176.4 (2)	C51—C52—C53—C54	−176.9 (2)
C24—C25—C26—C27	−175.7 (2)	C52—C53—C54—C55	179.5 (2)
C25—C26—C27—C28	−178.6 (3)	C53—C54—C55—C56	−177.6 (3)

### Hydrogen-bond geometry (Å, º)

**Table:**

*D*—H···*A*	*D*—H	H···*A*	*D*···*A*	*D*—H···*A*
O1—H1*O*···N1	0.73 (3)	2.13 (3)	2.684 (3)	135 (3)
O2—H2*O*···N3	0.78 (3)	2.09 (3)	2.685 (3)	133 (3)
O3—H3*O*···N5	0.84 (3)	1.91 (3)	2.662 (3)	148 (3)
O4—H4*O*···N7	0.75 (3)	2.00 (3)	2.663 (3)	146 (3)
N2—H2*N*···S7^i^	0.86 (3)	2.58 (3)	3.430 (3)	169 (3)
N4—H4*N*···S3^ii^	0.81 (3)	2.64 (3)	3.439 (3)	171 (2)
N6—H6*N*···S5^iii^	0.84 (3)	2.59 (3)	3.398 (3)	162 (2)
N8—H8*N*···S1^iv^	0.83 (3)	2.61 (3)	3.403 (3)	160 (2)

Symmetry codes: (i) *x*, −*y*+1, *z*+1/2; (ii) −*x*, −*y*+2, −*z*+2; (iii) −*x*+1, −*y*+2, −*z*+2; (iv) *x*, −*y*+1, *z*−1/2.
